# Association Between Probable Adult ADHD and Profession in India: A Cross-Sectional Study

**DOI:** 10.7759/cureus.112114

**Published:** 2026-07-05

**Authors:** Anirban Das Gupta, Biswabina Ray, Aniruddha Basu, Ritesh Singh, Satabdi Sarkar, Anasuya Ghosh

**Affiliations:** 1 Anatomy, All India Institute of Medical Sciences, Kalyani, Kalyani, IND; 2 Psychiatry, All India Institute of Medical Sciences, Kalyani, Kalyani, IND; 3 Community Medicine, All India Institute of Medical Sciences, Kalyani, Kalyani, IND

**Keywords:** adult attention deficit hyperactivity disorder (adhd), doctors, india, occupational health, professional groups, screening

## Abstract

Background: Attention-deficit/hyperactivity disorder (ADHD) is a neurodevelopmental disorder that may persist into adulthood. Adult ADHD remains understudied in India, particularly among professional groups.

Objectives: The objectives of the study are to determine the proportion of participants showing probable adult ADHD screening positivity through an online questionnaire and to compare screening positivity across various professional groups.

Methods: This cross-sectional study used an online questionnaire consisting of demographic information, associated factors, and the validated Adult ADHD Self-Report Scale (ASRS) Diagnostic and Statistical Manual of Mental Disorders, Fifth Edition (DSM-5) screening tool. The questionnaire was distributed via WhatsApp (Meta Platforms, Inc., Menlo Park, CA, USA) to various professional groups across India. A cut-off score of 14 was used to identify probable ADHD.

Results: Among 355 respondents, 115 (32.4%) showed probable adult ADHD screening positivity. Differences across professional groups were not statistically significant (p > 0.05). Screening positivity was significantly associated with marital status and work environment in unadjusted chi-squared analysis. Participants who screened positive reported higher screen time across professional categories than those who screened negative.

Conclusions: Probable adult ADHD screening positivity was observed across multiple professional groups and was not confined to a single occupation. The observed associations with relationship status and work environment should be interpreted as exploratory and non-causal. These findings support the need for workplace awareness, structured screening pathways, and further studies with clinical confirmation and multivariable analysis.

## Introduction

Attention-deficit/hyperactivity disorder (ADHD) is a neurodevelopmental disorder characterized by inattention and hyperactivity, typically arising during childhood. Contrary to earlier beliefs that ADHD is exclusively a childhood disorder, research has established that ADHD symptoms may persist into adulthood in a substantial proportion of individuals [1-3]. This recognition of ADHD as a lifelong condition prompted revisions in the most recent Diagnostic and Statistical Manual of Mental Disorders (DSM) criteria to facilitate diagnosis across the lifespan.

ADHD is increasingly recognized not as a simple behavioral disorder but as a complex syndrome affecting the brain's executive functions, including organization, attention to detail, alertness regulation, focus, working memory, motivation, and emotional management. These cognitive functions work together to complete various tasks, with individuals with ADHD showing substantial impairment in executive functions compared to their peers [4].

Though many individuals find their ADHD symptoms becoming less problematic with age due to brain maturation or changing environmental demands, significant impairment persists into adulthood for many. Research has shown that functional impairments can continue into middle age and beyond, with bodily changes potentially causing late-onset ADHD-like impairments. ADHD in adults can have a significant impact on physical and psychological health and is associated with social, occupational, and legal consequences [5].

ADHD significantly impacts educational attainment and employment stability, with studies indicating that only 50% of adults with ADHD can maintain full-time employment compared with 72% of adults without the disorder [6,7]. However, ADHD traits may also be associated with positive attributes such as cognitive flexibility, empathy, creativity, and entrepreneurial tendencies that favor self-employment [8,9]. A study by Lee et al. [10] examining famous people with potential ADHD found high skill levels in occupation, suggesting positive aspects of ADHD in adults.

The worldwide prevalence of ADHD varies considerably in the literature. A meta-analysis by Polanczyk et al. [11] found that geographic location plays a limited role in variability in prevalence estimates, which was primarily explained by methodological characteristics of studies. The WHO World Mental Health Survey Initiative found that an average of 3.5% of workers across 10 countries met criteria for adult ADHD [6], with significant workplace impairment associated with the condition. In India, a 2020 study reported ADHD prevalence at 0.3% (0.2%-0.5%) of the population [12].

Despite increasing awareness of adult ADHD globally, there is limited research on probable ADHD screening positivity among different professional groups in India. The present investigation screened for probable adult ADHD symptoms across various professional groups in India. The primary objective was to determine the proportion of respondents showing probable adult ADHD screening positivity using a validated screening questionnaire. Secondary objectives included identifying factors associated with screening positivity and comparing screening positivity across different professions. The study intended to assess whether probable adult ADHD screening positivity was associated with profession.

## Materials and methods

Study design and participants

A cross-sectional analytical study was conducted using an online questionnaire distributed via social media platforms. The questionnaire was circulated through WhatsApp groups (Meta Platforms, Inc., Menlo Park, CA, USA) representing various professions across India, with targeted efforts to include major professional groups. Data collection occurred over a six-month period, resulting in 355 responses.

The study was approved by the Institutional Ethics Committee, All India Institute of Medical Sciences (AIIMS), Bhubaneswar, under the title "Probable Adult Type of Attention Deficit and Hyperactive Disorder (ADHD) and Profession in India: Finding Association Through a Cross-Sectional Study." The present manuscript title was aligned with the ethics-approved title.

The sample size was estimated using the standard formula for prevalence studies, $n=Z^{2}p\left( 1-p \right)/d^{2}$, where *Z* = 1.96 for 95% confidence level, *p* = 20% based on the upper expected prevalence estimate from prior Indian literature, and *d* = 5% absolute precision. This yielded a minimum required sample of approximately 246 participants. The final analyzed sample included 355 respondents, which exceeded the calculated minimum sample size [13].

Questionnaire development

The questionnaire consisted of two sections. Section A collected demographic information and factors potentially associated with ADHD; this section was validated by five psychiatrists not affiliated with the institution where the study was initiated. Section B used the Adult ADHD Self-Report Scale (ASRS) DSM-5 Screening Tool developed by New York University and Harvard University; permission to use the instrument was obtained from the rights holder. The non-proprietary questionnaire items are provided in the appendix. The proprietary ASRS screener items are cited and described but not reproduced verbatim as per agreement.

The ASRS, originally described by Kessler et al., was used as the screening instrument [14]. The DSM-5 version of the WHO Adult ADHD Self-Report Screening Scale, as described by Ustun et al. [15], was used in the present study after obtaining appropriate permission from New York University and Harvard University. The ASRS is a validated instrument with reported sensitivity of 68.7% and specificity of 99.5% in the original short screener validation [14]. The updated DSM-5 version of the ASRS Screening Scale includes four questions based on DSM-5 criteria and two questions related to executive functioning impairments commonly seen in adults with ADHD [15,16]. A score of 14 or higher was used to define probable adult ADHD screening positivity, consistent with the DSM-5 ASRS screener scoring approach described by Ustun et al. This threshold was selected for screening purposes because it has been reported to provide favorable operating characteristics, but it was not used as a substitute for clinical diagnosis [15].

While the ASRS has been widely used across various cultural contexts [17-19], there is a potential need for cultural adaptation when used in diverse settings such as India. Regnart et al. [20] emphasized the importance of translating the ASRS into indigenous languages for better application in developing countries. However, given the English proficiency of the target population, which included professionals and students, the original English version of the scale was utilized. A pilot study with 25 participants was conducted to identify and address technical issues before final deployment.

Inclusion and exclusion criteria

Participants were required to be at least 18 years of age, residents of India, and willing to provide electronic consent. Responses from individuals not meeting these criteria were excluded from analysis.

Questionnaire submissions were screened before analysis. Responses were retained only when essential eligibility details and ASRS screening responses were available. Incomplete responses with missing eligibility information or missing ASRS data were excluded. Duplicate responses were checked using available identifiers such as email entry, response time stamp, and response pattern; when duplication was suspected, only one complete valid response was retained. Because the questionnaire link was circulated manually through professional WhatsApp groups rather than through a fully open public portal, the risk of bot responses was considered low; nevertheless, submissions were screened for implausible or repetitive entries before final analysis.

Data analysis

Descriptive statistics were calculated for demographic variables. Statistical analysis was performed using IBM SPSS Statistics for Windows, Version 19.0 (IBM Corp., Armonk, NY, USA). Chi-squared tests were used to examine associations between probable ADHD screening status and socio-demographic or work-related factors. The analyses were exploratory and unadjusted; no formal correction for multiple comparisons was applied. A cut-off score of 14 on the ASRS was used to identify individuals showing probable adult ADHD screening positivity. Percentages were calculated to one decimal place.

Professional categories were consolidated from open-ended responses according to the primary occupation reported by each participant. Business included respondents reporting self-owned or commercial business roles; students included those primarily identifying as students; health professionals included medical and allied health-related occupations; engineers included technical engineering roles; teachers included school, college, or academic teaching roles. Non-technical professionals included salaried or professional roles that were not primarily clinical, engineering, teaching, student, or business-based, such as administrative, managerial, clerical, finance, service-sector, and other office-based professional roles. This categorization was completed before analysis to allow comparison across broad occupational groups.

## Results

Demographic characteristics

The demographic and baseline characteristics of the respondents are summarized in Table 1. Of the 355 respondents, 227 (63.9%) were male, and the mean age was 34.2 years (SD = 12.2). Regarding marital status, 195 (54.9%) were married and 155 (43.7%) were single.

**Table 1:** Demographic and baseline characteristics of respondents

Characteristic	Category	n (%) or value
Total respondents	All participants	355 (100.0%)
Age	Mean (SD) (years)	34.2 (12.2)
Biological sex	Male	227 (63.9%)
	Female	128 (36.1%)
Marital status	Married	195 (54.9%)
	Single	155 (43.7%)
	Divorced or separated	4 (1.1%)
	Widowed	1 (0.3%)
Predominant nature of job	Indoor/sedentary	285 (80.3%)
Family environment during upbringing	Nuclear family	213 (60.0%)
	Extended family	118 (33.2%)
	Other family environment	24 (6.8%)
Current living arrangement	Nuclear-family setting	218 (61.4%)
	Single/shared/non-nuclear arrangement	137 (38.6%)
Relationship with near family members	Disturbed	30 (8.5%)
Relationship with co-workers	Disturbed	20 (5.6%)
Work environment	Predominantly non-friendly	39 (11.0%)
Current medical consultation/medication/counseling	Not receiving any consultation/medication/counseling	266 (74.9%)
Current treatment for mental health issues	Present	23 (6.5%)

Probable ADHD screening status by profession

Using a cut-off score of 14 on the ASRS, 115 (32.4%) respondents showed probable adult ADHD screening positivity and 240 (67.6%) screened negative. Table 2 presents probable ADHD screening status across professional categories. Differences across professional groups were not statistically significant (p > 0.05).

**Table 2:** ADHD screening status in relation to profession ADHD: attention-deficit/hyperactivity disorder

Profession	Screened positive n (%)	Screened negative n (%)	Total n (%)
Business	5 (23.8%)	16 (76.2%)	21 (100.0%)
Student	26 (36.6%)	45 (63.4%)	71 (100.0%)
Non-technical professionals	21 (42.0%)	29 (58.0%)	50 (100.0%)
Health professionals	41 (31.1%)	91 (68.9%)	132 (100.0%)
Engineer	9 (39.1%)	14 (60.9%)	23 (100.0%)
Teacher	13 (22.4%)	45 (77.6%)	58 (100.0%)
Total	115 (32.4%)	240 (67.6%)	355 (100.0%)

Factors associated with probable ADHD screening status

Associations between probable ADHD screening status and socio-demographic or work-related variables are presented in Table 3. Chi-squared statistics and p-values are shown in separate columns. Statistically significant unadjusted associations were observed for marital status and work environment.

**Table 3:** Association of ADHD screening status with socio-demographic and work-related factors Percentages are row-wise. Each variable is reconciled to the confirmed overall screening totals of 115 positive and 240 negative respondents. Chi-squared statistics and p-values are presented in separate columns. * indicates statistically significant p-value (p < 0.05).

Variable	Group	Screened positive n (%)	Screened negative n (%)	Chi-squared statistic	p-value
Biological sex	Male	76 (33.5%)	151 (66.5%)	0.339	0.560
	Female	39 (30.5%)	89 (69.5%)		
Marital status	Divorced or separated	2 (50.0%)	2 (50.0%)	9.924	0.019*
	Married	50 (25.6%)	145 (74.4%)		
	Single	63 (40.6%)	92 (59.4%)		
	Widowed	0 (0.0%)	1 (100.0%)		
Current family arrangement	Non-nuclear	18 (34.6%)	34 (65.4%)	7.618	0.055
	Nuclear	62 (28.4%)	156 (71.6%)		
	Shared accommodation	21 (50.0%)	21 (50.0%)		
	Single	14 (32.6%)	29 (67.4%)		
Relationship with near family member	Disturbed	11 (36.7%)	19 (63.3%)	0.273	0.601
	Not disturbed	104 (32.0%)	221 (68.0%)		
Occupation	Contractual government	7 (26.9%)	19 (73.1%)	8.062	0.089
	Regular government	30 (30.3%)	69 (69.7%)		
	Private	35 (36.5%)	61 (63.5%)		
	Self-employed	4 (12.9%)	27 (87.1%)		
	Unemployed/student	39 (37.9%)	64 (62.1%)		
Hours working per week	0-40	38 (29.0%)	93 (71.0%)	3.892	0.421
	41-45	27 (40.3%)	40 (59.7%)		
	46-50	21 (35.6%)	38 (64.4%)		
	51-60	14 (34.1%)	27 (65.9%)		
	>60	15 (26.3%)	42 (73.7%)		
Nature of job	Indoor	92 (32.3%)	193 (67.7%)	0.009	0.926
	Outdoor	23 (32.9%)	47 (67.1%)		
Relationship with co-workers	Disturbed	11 (55.0%)	9 (45.0%)	5.346	0.069
	Not disturbed	82 (30.3%)	189 (69.7%)		
	Not applicable	22 (34.4%)	42 (65.6%)		
Work environment	Predominantly friendly	74 (29.0%)	181 (71.0%)	11.640	0.003*
	Predominantly non-friendly	22 (56.4%)	17 (43.6%)		
	Not applicable	19 (31.1%)	42 (68.9%)		
Interpersonal verbal difference present	Always	1 (25.0%)	3 (75.0%)	9.307	0.097
	Often	9 (64.3%)	5 (35.7%)		
	Sometimes	19 (33.3%)	38 (66.7%)		
	Occasionally	38 (34.9%)	71 (65.1%)		
	Never	31 (25.8%)	89 (74.2%)		
	Not applicable	17 (33.3%)	34 (66.7%)		
Interpersonal physical difference present	Always	2 (40.0%)	3 (60.0%)	7.148	0.210
	Often	0 (0.0%)	4 (100.0%)		
	Sometimes	9 (56.2%)	7 (43.8%)		
	Occasionally	14 (37.8%)	23 (62.2%)		
	Never	72 (30.4%)	165 (69.6%)		
	Not applicable	18 (32.1%)	38 (67.9%)		
Sports	Predominantly indoor	36 (35.6%)	65 (64.4%)	0.726	0.696
	Predominantly outdoor	65 (31.4%)	142 (68.6%)		
	Not applicable	14 (29.8%)	33 (70.2%)		

Relationship between ADHD and interpersonal conflicts

Table 4 presents verbal and physical conflict patterns across professional groups by probable ADHD screening status. Verbal conflicts were more frequent among participants who screened positive in several professional categories, particularly teachers, health professionals, and business professionals. Physical conflicts were less frequent overall.

**Table 4:** Association of probable adult ADHD with verbal and physical conflict across professions NTP: non-technical professionals; ADHD: attention-deficit/hyperactivity disorder

Profession	ADHD status	Verbal conflict + n (%)	Verbal conflict - n (%)	Physical conflict + n (%)	Physical conflict - n (%)
Business	ADHD + (n = 5)	3 (60.0%)	2 (40.0%)	2 (40.0%)	3 (60.0%)
	ADHD - (n = 16)	7 (43.8%)	9 (56.2%)	0 (0.0%)	16 (100.0%)
Student	ADHD + (n = 26)	6 (23.1%)	20 (76.9%)	1 (3.8%)	25 (96.2%)
	ADHD - (n = 45)	17 (37.8%)	28 (62.2%)	3 (6.7%)	42 (93.3%)
NTP	ADHD + (n = 21)	11 (52.4%)	10 (47.6%)	2 (9.5%)	19 (90.5%)
	ADHD - (n = 29)	14 (48.3%)	15 (51.7%)	1 (3.4%)	28 (96.6%)
Health professionals	ADHD + (n = 41)	29 (70.7%)	12 (29.3%)	3 (7.3%)	38 (92.7%)
	ADHD - (n = 91)	51 (56.0%)	40 (44.0%)	6 (6.6%)	85 (93.4%)
Engineer	ADHD + (n = 9)	6 (66.7%)	3 (33.3%)	1 (11.1%)	8 (88.9%)
	ADHD - (n = 14)	1 (7.1%)	13 (92.9%)	1 (7.1%)	13 (92.9%)
Teacher	ADHD + (n = 13)	12 (92.3%)	1 (7.7%)	2 (15.4%)	11 (84.6%)
	ADHD - (n = 45)	20 (44.4%)	25 (55.6%)	3 (6.7%)	42 (93.3%)

Screen time and ADHD

Table 5 presents the proportion of participants reporting daily screen time greater than five hours across professional categories by probable ADHD screening status. Participants who screened positive for probable ADHD showed higher screen time in each professional category than those who screened negative.

**Table 5:** Daily screen time greater than five hours in relation to probable ADHD screening status ADHD: attention-deficit/hyperactivity disorder

Profession	Screened positive n/N (%)	Screened negative n/N (%)
Business	1/5 (20.0%)	1/16 (6.2%)
Engineer	5/9 (55.6%)	6/14 (42.9%)
Teacher	2/13 (15.4%)	4/45 (8.9%)
Non-technical professionals	6/21 (28.6%)	3/29 (10.3%)
Student	4/26 (15.4%)	4/45 (8.9%)
Health professionals	4/41 (9.8%)	2/91 (2.2%)

Financial status and ADHD

Table 6 presents the distribution of participants reporting monthly income below INR 50,000 across professional categories by probable ADHD screening status. Lower income was more common among participants who screened positive for probable ADHD across professional categories.

**Table 6:** Monthly income below INR 50,000 in relation to probable ADHD screening status ADHD: attention-deficit/hyperactivity disorder

Profession	Screened positive n/N (%)	Screened negative n/N (%)
Teachers	8/13 (61.5%)	11/45 (24.4%)
Non-technical professionals	16/21 (76.2%)	13/29 (44.8%)
Health professionals	27/41 (65.9%)	35/91 (38.5%)
Engineers	6/9 (66.7%)	3/14 (21.4%)
Business	5/5 (100.0%)	7/16 (43.8%)

## Discussion

This study found that 115 (32.4%) respondents showed probable adult ADHD screening positivity, with no statistically significant differences across professional groups. This value should be interpreted strictly as screening positivity rather than diagnostic prevalence, because no structured clinical diagnostic interview was performed. Therefore, the findings indicate the proportion of respondents who screened positive on the ASRS DSM-5 tool, not the confirmed prevalence of adult ADHD.

The observed screening positivity of 32.4% is considerably higher than most epidemiological estimates of adult ADHD. This difference may be explained by the use of a screening tool rather than clinical diagnosis, voluntary online participation, possible self-selection by participants with relevant symptoms, and the characteristics of an educated professional sample. The findings align more closely with Mattos et al. [17], who reported a high screening rate among medical students, although only a smaller proportion received a confirmed ADHD diagnosis upon clinical interview. Similarly, Shi et al. [18] reported likely ADHD cases after clinical evaluation among Chinese medical students. This discrepancy between screening positivity and confirmed diagnosis is important when interpreting the present findings, as the true prevalence of diagnosable ADHD in this sample remains uncertain without clinical follow-up.

The absence of a significant gender association (p = 0.560) suggests that probable adult ADHD screening positivity in this sample was not limited to one sex. However, this observation must be interpreted cautiously because the study was based on voluntary online screening and unadjusted analysis.

The absence of significant differences in probable ADHD screening positivity across professions suggests that symptoms may transcend occupational boundaries. These findings differ somewhat from Pritesh et al.'s study, which found a statistically significant difference in ADHD prevalence by educational program, with MBBS students showing the highest prevalence, followed by physiotherapy students, while no nursing students reported ADHD symptoms [13]. International research by Verheul et al. [21] suggested that certain professions or work arrangements, such as self-employment, may be more compatible with ADHD traits, which could explain some of the variation observed across professional categories, although these differences did not reach statistical significance in the present study.

Two statistically significant unadjusted associations emerged with probable ADHD screening status: marital status (p = 0.019) and work environment (p = 0.003). Single individuals were more likely to screen positive, suggesting that relationship context may be relevant to the expression or reporting of ADHD symptoms. Non-friendly work environments also showed an association with screening positivity, with specific workplace factors affecting several ADHD symptom components. Because multiple comparisons were performed without formal correction, these associations should be considered exploratory and interpreted with caution.

Significant associations between childhood family environment and specific symptoms, including difficulty relaxing, interrupting conversations, and dependence on others, align with developmental trajectory models of ADHD [1-3]. This highlights the developmental context of ADHD and suggests that family structure may influence the expression of specific ADHD symptoms.

This study revealed higher rates of verbal conflicts among individuals who screened positive for probable ADHD across professions, particularly among teachers, health professionals, and business professionals, as shown in Table 4. This pattern across diverse occupational settings suggests that probable ADHD symptoms may be associated with greater difficulty in managing interpersonal conflicts across professional contexts.

Associations between co-worker relations and multiple ADHD features underscore the interpersonal dimension of workplace functioning. Although the overall association with co-worker relationship status did not reach statistical significance (p = 0.069), the pattern supports the need to examine workplace relationships in future research.

A pattern of lower income below INR 50,000 monthly was observed among respondents who screened positive for probable ADHD across professional categories. The detailed values are presented in Table 6. This finding suggests a correlation between probable ADHD screening positivity and income-related outcomes but does not establish causality. Other confounding variables, including age, career stage, employment type, educational level, and co-morbid psychiatric symptoms, may influence this observation.

Individuals with probable ADHD screening positivity reported higher daily screen time greater than five hours across professional categories, a pattern consistent with previous reports linking problematic internet use and ADHD symptoms [22]. This observation should be interpreted cautiously because the study did not establish the direction of association between screen time and ADHD symptoms.

Although not statistically significant, the distribution of screening results among respondents who participated in indoor or outdoor games suggested a possible protective pattern. This observation should be explored in future studies with objective measures of physical activity.

The presence of probable ADHD screening positivity among various professions resonates with research by Lee et al. [10] and de Schipper et al. [23], which identified positive attributes associated with ADHD, including creativity, high energy levels, and flexibility. Workplace interventions should leverage these strengths while addressing challenges.

These findings emphasize the need for ADHD awareness and supportive workplace approaches across Indian professional settings. However, the results should be interpreted as exploratory screening findings rather than evidence of diagnostic prevalence or causal relationships. Larger studies using clinical confirmation and adjusted statistical modeling are needed before firm occupational or workplace conclusions can be drawn.

Limitations

This study has important limitations. It used a voluntary online survey, which may have introduced selection bias and may not represent all Indian professional groups. The ASRS is a screening tool and cannot substitute for a structured clinical diagnostic assessment; therefore, the findings indicate probable ADHD screening positivity rather than confirmed ADHD. The use of the English version of the tool among educated professionals may limit generalizability to populations with different linguistic backgrounds. The broad grouping of professions, absence of adjustment for potential confounders, lack of formal correction for multiple comparisons, and cross-sectional design limit causal interpretation. Although variables such as age, education, and self-reported treatment for mental health conditions were collected, multivariate analysis was not performed in the present exploratory analysis because of subgroup size limitations and sparse cells in several categories. Future studies should use culturally adapted tools, clinical confirmation, longitudinal designs, and multivariable regression models adjusting for age, sex, education, income, career stage, and co-morbid psychiatric variables. Future community-based studies using representative sampling and structured clinical diagnostic interviews are required to confirm these observations.

## Conclusions

Probable adult ADHD screening positivity was observed across diverse professional groups in this online sample. The observed associations with relationship status and work environment were based on exploratory unadjusted analyses and should not be interpreted as causal. These findings support the need for awareness and structured screening pathways, but further studies using clinical confirmation and multivariable analysis are required before firm conclusions can be made.

## Appendices

Study questionnaire in English (non-proprietary items)

The questionnaire used in the study had two parts. Part A collected demographic, occupational, lifestyle, relationship, and medical condition information. Part B used the ASRS DSM-5 Screening Tool under permission from NYU and Harvard University. Because the ASRS screener is proprietary, the ASRS item text is cited and described in the Materials and Methods section and is not reproduced verbatim here because of copyright restrictions.

**Table 7:** Non-proprietary questionnaire items ASRS: Adult ADHD Self-Report Scale; DSM-5: Diagnostic and Statistical Manual of Mental Disorders, Fifth Edition

Item no.	Variable/question domain	Response options/notes
1	Email address	Free-text response
2	Consent to participate	Agree/disagree
3	Age in years	Free-text numeric response
4	Biological sex/gender	Female/male/other
5	Marital status	Single/married/divorced or separated/widowed
6	Family environment during upbringing	Nuclear family/extended family/childless family/grandparent family/step family/single-parent family
7	Current living arrangement	Single/nuclear family/non-nuclear living arrangement/shared accommodation
8	Relation with near family members	Disturbed/not disturbed
9	Average daily time spent playing video games	Hours per day; zero if not applicable
10	Average daily time spent watching entertainment videos	Hours per day; zero if not applicable
11	Average daily computer use for professional work	Hours per day; zero if not applicable
12	Highest educational qualification	Matriculation/higher secondary/graduation/postgraduation/doctorate/none of the above
13	Major profession	Free-text response
14	Type of job	Government/private/contractual government/self-employed
15	Monthly salary	Free-text or category response as used in the survey
16	Years in current profession	Free-text numeric response
17	Working hours per week	40/45/50/60/more than 60 hours
18	Predominant nature of job	Indoor/outdoor
19	Cigarette use per day	Number per day; zero if none
20	Alcohol use per week	Frequency per week; zero if none
21	Age at starting smoking	Age in years; zero if not applicable
22	Age at starting alcohol use	Age in years; zero if not applicable
23	Age at starting chewing gutkha	Age in years; zero if not applicable
24	Usual relation with most co-workers	Disturbed/not disturbed/not applicable
25	Work environment	Predominantly friendly/predominantly non-friendly/not applicable
26	Interpersonal differences escalating to verbal conflicts among co-workers	Never/occasionally/often/sometimes/always/not applicable
27	Interpersonal differences escalating to physical tussle among co-workers	Always/often/sometimes/rarely/never/not applicable
28	Preferred sports type	Predominantly indoor games/predominantly outdoor games/not applicable
29a	Current consultation, medication, or counseling for hypertension	Yes/no
29b	Current consultation, medication, or counseling for diabetes	Yes/no
29c	Current consultation, medication, or counseling for depression	Yes/no
29d	Current consultation, medication, or counseling for anxiety	Yes/no
29e	Current consultation, medication, or counseling for hyperthyroidism	Yes/no
29f	Current consultation, medication, or counseling for hypothyroidism	Yes/no
29g	Current consultation, medication, or counseling for mood disorder	Yes/no
29h	Current consultation, medication, or counseling for eating disorder	Yes/no
29i	Current consultation, medication, or counseling for suicidal tendency	Yes/no
29j	No current consultation, medication, or counseling for the listed conditions	Yes/no
30a	Entitled to take leave	Yes/no/not applicable
30b	Spouse working	Yes/no/not applicable
30c	Other family member staying with participant is working	Yes/no/not applicable
30d	Job entails night shift	Yes/no/not applicable
30e	Work necessitates emergency duty	Yes/no/not applicable
30f	Involvement in gambling	Yes/no/not applicable
31	ASRS DSM-5 screener	Six-item proprietary ASRS DSM-5 screener; response options: never/rarely/sometimes/often/very often
